# Significance of Right Ventricular Dysfunction in Predicting Short-Term Survival Among Patients With Sepsis and Septic Shock: A Prognostic Analysis

**DOI:** 10.1155/ccrp/5511135

**Published:** 2025-03-14

**Authors:** Sukrisd Koowattanatianchai, Patchara Kochaiyapatana, Narueporn Eungsuwat, Vimonsri Rangsrisaeneepitak, Katkanit Thammakumpee, Kiraphol Kaladee

**Affiliations:** ^1^Division of Cardiology, Department of Medicine, Burapha Hospital, Burapha University, Chonburi, Thailand; ^2^Department of Medicine, Burapha Hospital, Burapha University, Chonburi, Thailand; ^3^Division of Endocrinology and Metabolism, Department of Medicine, Burapha University, Chonburi, Thailand; ^4^School of Health Science, Sukhothai Thammathirat Open University, Nonthaburi, Thailand

**Keywords:** echocardiography, right ventricular dysfunction, sepsis, septic shock

## Abstract

**Objective:** This study sought to evaluate the association between right ventricular (RV) dysfunction and short-term in-hospital mortality among patients with sepsis and septic shock.

**Methods:** A prospective cohort study was conducted on adult patients admitted at Burapha University Hospital for sepsis and septic shock from October 1, 2022, through June 30, 2023, who underwent echocardiography within 72 h after admission. RV dysfunction and other echocardiographic findings were analyzed and defined using the American Society of Echocardiography criteria. The primary outcome examined in this study was 28-day in-hospital mortality. Secondary outcomes included maximal blood lactate levels, length of intensive care unit (ICU) stay, and duration of mechanical ventilation.

**Results:** A total of 104 patients (mean age: 69.54 ± 14.88 years) were enrolled in this study. Among the included patients, 32 (30.8%) developed septic shock whereas 20 (19.2%) exhibited RV dysfunction. Cox regression analysis showed that patients with RV dysfunction had a 28-day in-hospital mortality rate 5.53 times higher than that of patients with normal RV function (95% confidence intervals: 1.98–15.42; *p*=0.001). Regarding the secondary outcomes, patients with RV dysfunction exhibited a significantly higher mean serum lactate level (5.72 ± 4.96 vs. 3.74 ± 3.29 mmol/L; *p*=0.034) and length of ICU stay (6.50 ± 2.86 vs. 2.84 ± 1.56 days; *p*=0.020) than did those with normal RV function.

**Conclusions:** RV dysfunction was associated with increased short-term mortality among patients with sepsis and septic shock. Assessing RV function among these patients facilitates precise prognostication and aids in guiding treatment strategies aimed at reducing mortality.

**Trial Registration:** ClinicalTrials.gov identifier: NCT06193109

## 1. Introduction

Sepsis, which is commonly encountered in clinical practice, carries considerable clinical significance given its potential for progression to septic shock, which can cause organ dysfunction, making it the leading cause of death among critically ill patients worldwide [1]. In cases with severe sepsis and septic shock, mortality rates can reach as high as approximately 50%–80%, highlighting the significance of sepsis as a global public health challenge [1–3].

Among the affected organs, the heart has emerged as a significant target, with notable implications including left ventricular (LV) dysfunction, including systolic and/or diastolic dysfunction, typically assessed using echocardiography. This condition is commonly referred to as “septic cardiomyopathy” (SCM) [4]. Aside from LV dysfunction, the prognostic impact of right ventricular (RV) dysfunction in patients with septic shock has garnered renewed interest [5–7]. However, prior research has primarily utilized a retrospective methodology and often lack essential parameters for evaluating RV function. Moreover, the prognostic impact of RV dysfunction on short-term mortality rates has remained a contentious topic of discussion.

Although a meta-analysis has established a significant association between the presence of RV dysfunction among patients with sepsis and septic shock and both short- and long-term mortality, the majority of studies have mainly compiled observational data [8]. Therefore, we aimed to conduct a prospective study to clarify the prognostic significance of RV function on 28-day in-hospital mortality among patients with sepsis and septic shock.

## 2. Materials and Methods

### 2.1. Study Design and Patients

A prospective study was conducted on hospitalized patients diagnosed with sepsis and septic shock at Burapha University Hospital, Chonburi province, Thailand, from October 1, 2022, through June 30, 2023. Burapha University Hospital is a regional, internationally accredited medical facility, which adheres to global medical school standards. Compared with other institutions, particularly tertiary hospitals, it demonstrates comparable attributes, including healthcare resources, medical staff, and standardized protocols. This is attributable to its capability of providing tertiary care and achieving quality certification per Healthcare Accreditation standards. Sepsis [9] was defined as a life-threatening organ dysfunction caused by a dysregulated host response to infection. Organ dysfunction can be identified as an acute change (≥ 2 points) in the total Sequential Organ Failure Assessment (SOFA) score as a consequence of infection. Septic shock [9] was defined as a subset of sepsis in which underlying circulatory and cellular/metabolic abnormalities are profound enough to substantially increase the risk of mortality. Septic shock can be identified as a clinical construct of sepsis with persisting hypotension requiring vasopressors to maintain a mean arterial pressure of ≥ 65 mmHg and serum lactate levels > 2 mmol/L (18 mg/dL) despite adequate volume resuscitation. The inclusion criteria comprised age of 18 years or older and a diagnosis of sepsis or septic shock according to the Sepsis-3 criteria [9]. All patients underwent echocardiography within 72 h after admission. The exclusion criteria were as follows: (1) prior diagnosis of pulmonary hypertension, (2) Cor Pulmonale, (3) significant valvular heart disease, (4) acute coronary syndrome, (5) aortic dissection, (6) heart failure, (7) inability to comply with follow-up within 28 days, and (8) patients with poor-quality echocardiography images and measurements. The present study was approved by the BUU Ethics Institutional Review Board (HS 114/2565) on March 29, 2023, and complies with the ethical standards of the Declaration of Helsinki.

### 2.2. Clinical Data Collection

All demographic, clinical, and laboratory data, as well as treatment information—including age, sex, body mass index, comorbidities, admission location, source of sepsis, incidence of septic shock, mechanical ventilation, acute respiratory distress syndrome (ARDS), acute kidney injury, use of hydrocortisone, use of inotropic drugs, intravenous fluids, blood urea nitrogen, creatinine, electrolytes, anion gap, pH, maximal lactate levels, cortisol levels, high-sensitivity troponin, albumin, and complete blood count—were prospectively collected. Estimated glomerular filtration rate (eGFR) was calculated according to the Chronic Kidney Disease Epidemiology Collaboration formula [10]. SOFA score and Acute Physiology and Chronic Health Evaluation (APACHE)-II scores were calculated on the day of admission. Mortality data throughout the 28-day hospitalization period were collected from the hospital's registry.

### 2.3. Echocardiography

Patients underwent transthoracic echocardiography examination using the Philips EPIQ CVx with the S5-1 sector array and the 3D X3-1 xMatrix array transducers (EPIQx3D, Philip Ultrasound, Inc., Bothell, USA). The examination was performed within 72 h of admission. All echocardiography examinations were performed by two experienced technician sonographers. American Society of Echocardiography (ASE) criteria [11] were utilized for echocardiographic assessment. New onset RV dysfunction was assessed using multimodality parameters as defined based on the ASE criteria (that is, tricuspid annular plane systolic excursion [TAPSE] < 16 mm, tissue Doppler-derived tricuspid lateral annular systolic velocity [S′] < 10 cm/s, RV fractional area change (RV FAC) < 35%). Data were analyzed by two experienced cardiologists (SK and PK) who were blinded to the patients' treatment. Any differences in interpretation and measurements were resolved by agreement. Herein, the acquisition time for the transthoracic echocardiography was 16.61 ± 13.54 h, with a minimum of 4 h and a maximum of 62 h after the diagnosis of sepsis or initial resuscitation.

### 2.4. Statistical Analysis

Continuous data were presented as median with the interquartile range (IQR), whereas categorical data were presented as numbers with their respective percentages. Continuous and categorical data were assessed using the Mann–Whitney *U* test and Fisher's exact test, respectively. In both univariate and multivariate analyses, categorical variables were reported as odds ratio (OR) along with their corresponding 95% confidence intervals (CIs). The investigation further included an analysis of patient survival at 28 days, employing cumulative time-to-event distribution (survival) demonstrated via Kaplan–Meier survival curves. The primary aim was to determine survival rate discrepancies between two distinct cohorts: one afflicted with RV dysfunction and the other without such dysfunction. This comparative analysis was executed using the log-rank test. Moreover, the impact of RV dysfunction on 28-day in-hospital mortality was explored through Cox proportional hazards regression analyses. The outcomes were reported using hazard ratios (HRs) accompanied by their respective 95% CIs, as well as adjusted HRs with their corresponding 95% CIs. A *p* value below 0.05 indicated statistical significance.

## 3. Results

### 3.1. Baseline Clinical Characteristics

**Figure 1:**
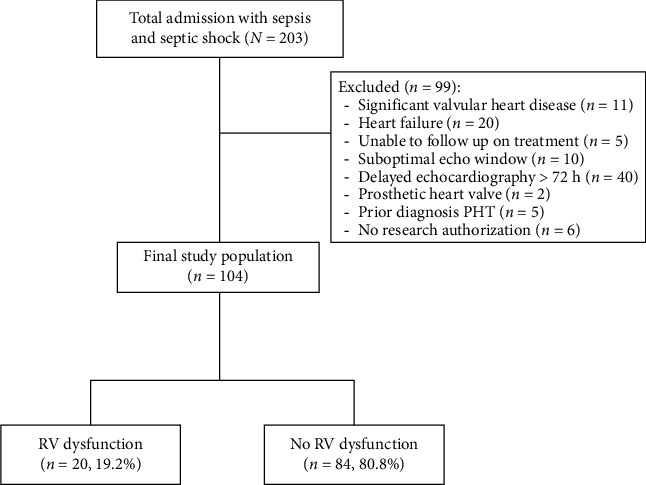
Study flow diagram.

A total of 203 patients diagnosed with sepsis and septic shock were admitted to Burapha University Hospital between October 1, 2022, and June 30, 2023. A total of 94 patients who failed to meet the inclusion criteria were excluded from the analysis (Figure 1). Consequently, 104 patients with a mean age of 69.54 ± 14.88 years were included in the study, among whom 58 (55.7%) were male. Among these patients, 32 (30.8%) were diagnosed with septic shock, whereas 20 (19.2%) exhibited RV dysfunction.

Respiratory system infections were most prevalent among the studied patients. Notably, individuals with RV dysfunction were more likely to be admitted to the intensive care unit (ICU), require mechanical ventilation, and develop ARDS than did those without RV dysfunction. Furthermore, the SOFA and APACHE II scores in patients with RV dysfunction were significantly higher than those with normal RV function (6.15 ± 4.08 vs. 4.73 ± 2.53, *p* = 0.05 and 18.1 ± 5.48 vs. 14.31 ± 5.32, *p* = 0.005, respectively; Table 1).

### 3.2. Laboratory Values

Laboratory results revealed a significant difference in pH levels between patients with RV dysfunction and those with normal RV function (7.31 ± 0.13 vs. 7.38 ± 0.11, *p* = 0.019). Specifically, the RV dysfunction group exhibited a higher degree of acidosis than did individuals with normal RV function. In addition, patients with RV dysfunction exhibited higher blood lactate levels than did individuals with normal RV function (5.72 ± 4.96 vs. 3.74 ± 3.29 mmol/L, *p* = 0.034; Table 2).

### 3.3. Echocardiographic Findings

Echocardiographic findings revealed that patients with RV dysfunction had a significantly lower LV ejection fraction than did those with normal RV function (50.99% ± 14.30% vs. 63.73% ± 10.09%, *p* < 0.001). In addition, patients with RV dysfunction tended to have pulmonary hypertension based on mean pulmonary arterial pressure measurements using Abbas's formula (34.74 ± 2.31 vs. 25.45 ± 6.61 mmHg, *p* = 0.006; Table 3).

### 3.4. Prognostic Factors and 28-Day In-Hospital Mortality

We then investigated the prognostic factors associated with 28-day in-hospital mortality in patients with sepsis and septic shock. During the initial univariable analysis, we found that several factors were significantly associated with 28-day in-hospital mortality, such as RV systolic dysfunction (HR: 7.86; *p* < 0.001), septic shock (HR: 2.60; *p* = 0.038), mechanical ventilation (HR: 4.68; *p* = 0.003), SOFA score (HR: 1.20; *p* = 0.021), and APACHE II score (HR: 1.09; *p* = 0.027) (Table 4).

Cox proportional hazards regression analysis was subsequently performed to identify and select clinically significant factors associated with 28-day in-hospital mortality. To control for confounding variables, RV dysfunction, septic shock, mechanical ventilation, SOFA score, APACHE II score, use of inotropic drugs, blood lactate levels, and diabetes were included. Finally, our analysis found that RV dysfunction was predominantly associated with 28-day in-hospital mortality (HR: 5.53; 95% CI: 1.98–15.42; and *p* = 0.001; Table 5).

**Figure 2:**
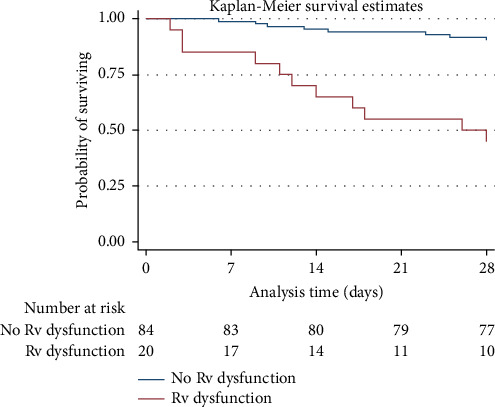
Kaplan–Meier survival curves.

In a cohort of 104 patients diagnosed with sepsis and septic shock followed over period of 2562 days, 19 mortalities were recorded. Among the cohort, 84 individuals exhibited normal RV function, with a mortality rate of 3.5 per 1000 person-days. In contrast, those with RV dysfunction exhibited a significantly higher mortality rate than did those with normal RV function (25.8 vs. 3.5 per 1000 person-days), with a mean survival duration of approximately 26 days as indicated by a 95% CI of 11–28 days. Notably, survival assessment at day 14 posttreatment, found that individuals with normal RV function exhibited a notably higher survival probability (0.95; 95% CI: 0.87–0.98) than did those with RV dysfunction (0.65; 95% CI: 0.40–0.82). Furthermore, at day 28 posttreatment, patients displaying normal RV function exhibited a higher survival probability (0.90; 95% CI: 0.81–0.95) than did those with RV dysfunction (0.45; 95% CI: 0.23–0.64). As depicted in the Kaplan–Meier survival curve, a significant difference in survival was observed between both groups (*p* < 0.001; Figure 2).

### 3.5. Secondary Outcomes Associated With RV Dysfunction

Regarding the secondary outcomes, which included serum lactate levels, length of ICU stay, and duration of mechanical ventilation, we observed significant differences in the mean serum lactate levels and length of ICU stay between patients with RV dysfunction and those with normal RV function (mean difference 1.98, 95% CI: 0.15–3.81; and *p* = 0.034 and mean difference 3.67, 95% CI: 0.58–6.75; and *p* = 0.020, respectively). In contrast, no significant difference in the duration of mechanical ventilation was found between both groups (mean difference 3.03, 95% CI: −0.15–6.20; and *p* = 0.062; Table 6).

## 4. Discussion

In clinical practice, physicians managing patients with severe sepsis who progress to septic shock often focus on assessing cardiac function, particularly LV function, while neglecting RV function. Consequently, the evaluation tends to be limited to the left ventricle. Thus, the present study aimed to evaluate the association between RV dysfunction and short-term in-hospital mortality among patients with sepsis and septic shock. Our results demonstrated that among patients with sepsis and septic shock, the presence of RV dysfunction increased the risk of 28-day in-hospital mortality. In addition, RV dysfunction was associated with elevated serum lactate levels and prolonged ICU stay, thereby increasing hospital costs.

Multiple factors are involved in the pathophysiology of RV dysfunction in sepsis and septic shock. However, the key mechanisms include direct compression of myocardial tissue, increased RV preload, and increased RV afterload [12]. Physicians typically prescribe intravenous fluids to patients with suspected sepsis, particularly in the early stages and when hypotension develops. These patients often receive aggressive fluid management to stabilize their condition. However, aggressive fluid administration increases RV preload, which may exacerbate the condition of patients with RV dysfunction. Despite this, the data presented herein indicate that patients with and without RV dysfunction received similar early interventions following admission, including fluid therapy, as reflected by the comparable volumes of intravenous fluids administered to both groups within the first 24 h (2626.85 ± 2062.63 mL vs. 2073.94 ± 1386.43 mL, *p*=0.151). These findings suggest that RV dysfunction may result from a combination of decreased RV contractility and increased RV afterload.

RV afterload may be aggravated by various factors, such as hypoxemia, hypercapnia, and acute respiratory failure [13]. Patients with sepsis who develop ARDS tend to have an increased risk of RV dysfunction due to both reduced RV contractility and increased RV afterload [12]. ARDS induces hypoxic pulmonary vasoconstriction and increased pulmonary pressure. An increased mean pulmonary arterial pressure is frequently associated with sepsis and ARDS through several mechanisms. These mechanisms include obstruction of the pulmonary microcirculation by microthrombi comprising platelets and leukocytes, active pulmonary vasoconstriction mediated by the autonomic nervous system, and the effects of hypoxia or vasoactive humoral mediators [14]. Moreover, the reduced production of nitric oxide, an endogenous vasodilator, in sepsis likely contributes to elevated pulmonary vascular resistance and pulmonary hypertension [15]. Our study found that patients with RV dysfunction had a higher incidence of ARDS and more frequent use of mechanical ventilation than did those with normal RV function. Moreover, echocardiography findings showed that patients with RV dysfunction exhibited higher mean pulmonary arterial pressure, as calculated by Abbas's formula, than did those with normal RV function. This increase in RV afterload leads to an uncoupling between pulmonary circulation and RV function. Owing to its limited capacity for effective contraction, RV lacks an adaptive reserve mechanism beyond dilation, which can displace the interventricular septum toward the LV, further reducing LV diastolic filling and stroke volume. These changes exacerbate LV compression, circulatory failure, and impaired oxygen delivery [16]. Moreover, tachycardia and increased stroke work elevate RV free wall tension, further increasing oxygen demand. Ultimately, cardiac output declines and results in systemic hypotension. RV ischemia occurs because of the combined effects of increased RV workload, elevated free wall tension, and reduced coronary perfusion. These mechanisms may induce more severe RV dysfunction [17].

From an echocardiographic viewpoint, the integrated data highlight that an increasing mean pulmonary arterial pressure is a pivotal factor in the pathophysiology of RV dysfunction, which subsequently declines RV function, as evidenced by reduced TAPSE, S′, and RV FAC. Concerning sepsis, the management of RV dysfunction should prioritize judicious fluid administration and monitoring for signs of RV dysfunction, particularly through clinical assessment and echocardiographic evaluation. If RV dysfunction is detected, fluid therapy should be guided by central venous pressure measurements [18]. The initiation of vasoactive support is essential considering it enhances RV contractility and prevents hemodynamic instability. Hypotension can precipitate RV ischemia, impairing RV function and potentially resulting in a self-perpetuating vicious cycle [19]. In addition, addressing reversible causes of increased pulmonary vascular resistance—such as hypoxia, acidosis, and lung hyperinflation associated with ARDS—through lung-protective mechanical ventilation strategies (e.g., low tidal volume, optimal positive end-expiratory pressure [PEEP], and maintaining plateau pressure below 30 mm Hg) may mitigate RV dysfunction [19].

The present prospective study also revealed that 19.2% of the patients diagnosed with sepsis and septic shock had RV dysfunction. Multiple studies have reported incidence rates of isolated RV dysfunction ranging from 10% to 50% [6, 8, 20]. Moreover, our study found that patients with RV dysfunction exhibited reduced LV contraction compared with those with normal RV function. Approximately 50% of the patients with severe sepsis and septic shock present with concomitant LV systolic dysfunction [21]. The current study further demonstrated that patients with RV dysfunction tended to have higher rates of septic shock. Thus, they required more frequent use of inotropic drugs than did those with normal RV function.

Moreover, patients with RV dysfunction exhibited an approximately five times higher risk of 28-day in-hospital mortality than did those with normal RV function. These findings are consistent with those presented in previous studies [22–25]. For instance, Bendary et al. [24] reported that RV dysfunction in patients with sepsis and septic shock was a significant independent predictor of 30-day mortality (OR: 2.01; 95% CI: 1.07–3.81; and *p*=0.031). Similarly, the multivariable regression model created by Lanspa et al. [25] demonstrated that RV dysfunction was associated with increased 28-day mortality (OR: 3.4; 95% CI: 1.7–6.8; and *p*=0.001) among patients with early sepsis and septic shock.

Furthermore, several studies and meta-analyses have consistently documented a correlation between RV dysfunction and long-term mortality [5, 7, 8, 26]. The persistence of RV dysfunction in sepsis appears to be associated with an increased risk of mortality. However, survivors of sepsis typically experience normalization of RV dysfunction after 7–14 days [21]. Thus, RV dysfunction may have a significantly greater effect on short-term rather than long-term mortality. The current study found that RV dysfunction was associated with greater illness severity, as indicated by higher SOFA and APACHE II scores. In addition, patients with RV dysfunction were more frequently prescribed mechanical ventilation, admitted to the ICU, and diagnosed with ARDS than were those with normal RV function. These factors may have contributed to the increased mortality rate observed in patients with RV dysfunction.

Evaluation of secondary outcomes, including serum lactate levels, length of ICU stay, and duration of mechanical ventilation, found that patients with RV dysfunction tended to have higher serum lactate levels and longer ICU stays than did those with normal RV function. Serum lactate serves as a marker of severity and prognosis in sepsis. Elevated lactate levels can be considered an early warning signal for organ dysfunction and have been identified as an independent prognostic predictor of mortality in patients with sepsis [27]. In addition, a prolonged ICU stay has been associated with increased long-term mortality [28].

Multiple parameters have been used to evaluate RV systolic function, including the RV index of myocardial performance (RIMP), TAPSE, RV FAC, S′, three-dimensional echocardiographic ejection fraction, and longitudinal strain and strain rate measured by Doppler tissue imaging and two-dimensional speckle tracking echocardiography (2D STE) [29]. In the current study, RV function in patients with sepsis and septic shock was assessed using TAPSE, S′, and RV FAC via transthoracic echocardiography (TTE). The parameters were selected owing to their established prognostic value, feasibility, and practicality in clinical practice. Both TAPSE and S′ have been validated against radionuclide ejection fraction. Meanwhile RV FAC, which reflects both longitudinal and radial components of RV contraction, has been found to be correlated with RV ejection fraction as measured by cardiac magnetic resonance (CMR) [30]. Currently, 2D STE is a relatively novel and sensitive method for assessing ventricular function, potentially revealing systolic dysfunction not detectable with conventional echocardiography. However, this method is time-consuming and requires well-trained echocardiographers and adequate image quality. In addition, it can be particularly challenging to implement in mechanically ventilated patients in an ICU setting [26]. The gold standard approach for measuring RV systolic function is CMR [31], which provides the most accurate and reproducible assessment of RV volumes, mass, and ejection fraction. However, in cases where patients experience hemodynamic instability or compromised vital signs, transporting these individuals for CMR assessments is not feasible. Consequently, TTE remains the primary modality for evaluating RV function in individuals diagnosed with sepsis or septic shock.

The findings presented in the current study further suggests that RV dysfunction in patients with sepsis and septic shock is an independent predictor of short-term mortality. Therefore, in such patients, physicians should assess RV function using noninvasive strategies, particularly transthoracic echocardiography, to enable early detection and guide treatment. This is especially important in patients with uncertain intravascular fluid status, as highlighted in the pathophysiology of RV dysfunction.

Some limitations of this study warranted discussion. First, this study utilized TTE to assess RV function in patients with sepsis and septic shock, the results of which may be affected by operator variability. CMR provides the highest accuracy for evaluating RV function in this patient population. Future research employing CMR to assess RV function in sepsis and septic shock could enhance the precision of these findings. In addition, the size of patient population in this study may limit some of the generalizability. Hence, further study with larger patient population can augment the study generalizability and applicability of the study findings.

## 5. Conclusion

The current study found that RV dysfunction was associated with an increased risk of 28-day in-hospital mortality among patients with sepsis and septic shock. The assessment of RV dysfunction using TTE can assist with identifying short-term prognosis and guiding the therapeutic management of these patients.

## Figures and Tables

**Table 1:** Baseline clinical characteristics.

Parameter	Total (*n* = 104)	RV dysfunction (*n* = 20)	No RV dysfunction (*n* = 84)	*p* value
Age (years), mean ± SD	69.54 ± 14.88	66.1 ± 14.30	70.36 ± 17.04	0.252
Male, *n* (%)	58 (55.8)	11 (55.0)	47 (55.9)	0.999
BMI (kg/m^2^), mean ± SD	24.49 ± 5.36	24.77 ± 6.43	24.42 ± 5.11	0.799
DM, *n* (%)	55 (52.9)	9 (45.0)	46 (54.8)	0.464
DLP, *n* (%)	50 (48.1)	10 (50.0)	40 (47.6)	0.999
Stroke, *n* (%)	15 (14.4)	3 (15.0)	12 (14.3)	0.999
HTN, *n* (%)	73 (70.2)	12 (60.0)	61 (72.6)	0.286
Asthma/COPD, *n* (%)	4 (3.9)	0	4 (4.76)	0.999
CAD/prior MI, *n* (%)	9 (8.7)	2 (10.0)	7 (8.3)	0.683
CKD, *n* (%)	28 (26.9)	5 (25.0)	23 (27.4)	0.999
Cirrhosis, *n* (%)	9 (8.7)	0	9 (10.7)	0.201
Admitting location, *n* (%)				0.001
ICU medicine	32 (30.8)	12 (60.0)	20 (23.8)	
ICU surgery	10 (9.6)	4 (20.0)	6 (7.1)	
Ward medicine	57 (54.8)	4 (20.0)	53 (63.1)	
Ward surgery	5 (4.8)	0	5 (6.0)	
Source of sepsis, *n* (%)				0.203
Respiratory	37 (35.6)	9 (45.0)	28 (33.3)	
Abdomen	25 (24.0)	4 (20.0)	21 (25.0)	
Genitourinary	19 (18.3)	1 (5.0)	18 (21.4)	
Skin/soft tissue	9 (8.7)	4 (20.0)	5 (6.0)	
Infective endocarditis	1 (1.0)	0 (0.0)	1 (1.2)	
Others	13 (12.5)	2 (10.0)	11 (13.1)	
Septic shock, *n* (%)	32 (30.8)	9 (45.0)	23 (27.4)	0.177
Mechanical ventilator, *n* (%)	42 (40.4)	15 (75.0)	27 (32.1)	0.001
PEEP, mean ± SD	5.37 ± 1.29	5.67 ± 1.40	5.21 ± 1.23	0.279
FiO_2_, mean ± SD	0.48 ± 0.18	0.55 ± 0.21	0.43 ± 0.16	0.046
ARDS, *n* (%)	4 (3.9)	4 (20.0)	0	0.001
Acute kidney injury, *n* (%)	60 (57.8)	13 (65.0)	47 (55.9)	0.616
Hydrocortisone usage, *n* (%)	15 (14.4)	7 (35.0)	8 (9.5)	0.008
SOFA score, mean ± SD	5.0 ± 2.92	6.15 ± 4.08	4.73 ± 2.53	0.050
APACHE II score, mean ± SD	15.04 ± 5.53	18.1 ± 5.48	14.31 ± 5.32	0.005
IV fluid in 24 h, mean ± SD	2180.27 ± 1543.27	2626.85 ± 2062.63	2073.94 ± 1386.43	0.151
Use of inotropic drugs, *n* (%)	29 (27.9)	9 (45.0)	20 (23.8)	0.093
Type of inotropic				0.051
None	74 (71.2)	11 (55.0)	63 (75.0)	
NE + dopamine	1 (0.9)	1 (5.0)	0	
NE	29 (27.9)	8 (40.0)	21 (25.0)	

Abbreviations: APACHE II, Acute Physiology and Chronic Health Evaluation II; ARDS, acute respiratory distress syndrome; BMI, body mass index; CAD, coronary artery disease; CKD, chronic kidney disease; COPD, chronic obstructive pulmonary disease; DLP, dyslipidemia; DM, diabetes mellitus; HTN, hypertension; ICU, intensive care unit; IV, intravenous; MI, myocardial infarction; NE, norepinephrine; PEEP, positive end-expiratory pressure; SOFA, Sequential Organ Failure Assessment.

**Table 2:** Laboratory values.

Parameters	Mean ± SD			*p* value
	Total (*n* = 104)	RV dysfunction (*n* = 20)	No RV dysfunction (*n* = 84)	
BUN (mg/dL)	34.93 ± 24.46	30.98 ± 23.95	35.87 ± 24.62	0.424
Serum creatinine (mg/dL)	2.49 ± 2.28	2.20 ± 1.94	2.56 ± 2.36	0.533
eGFR (mL/m^2^)	44.36 ± 30.77	45.92 ± 27.73	43.99 ± 31.59	0.803
Sodium (mmol/L)	134.40 ± 6.59	135.85 ± 8.86	134.06 ± 5.95	0.277
Potassium (mmol/L)	4.14 ± 0.75	4.23 ± 0.54	4.15 ± 0.73	0.899
Chloride (mmol/L)	99.38 ± 7.64	100.20 ± 9.59	99.18 ± 7.15	0.593
Bicarbonate (mmol/L)	20.44 ± 5.09	19.00 ± 6.10	20.79 ± 4.79	0.159
Anion gap	14.59 ± 6.32	16.65 ± 7.77	14.10 ± 5.88	0.105
pH	7.37 ± 0.12	7.31 ± 0.13	7.38 ± 0.11	0.019
pCO_2_ (mmHg)	32.48 ± 10.30	31.54 ± 14.77	32.72 ± 8.92	0.648
Maximal lactate (mmol/L)	4.14 ± 3.75	5.72 ± 4.96	3.74 ± 3.29	0.034
Cortisol (μg/dL)	22.40 ± 15.28	23.46 ± 18.04	21.76 ± 14.07	0.812
High sense troponin (ng/mL)	276.12 ± 537.68	647.55 ± 1094.75	188.73 ± 304.68	0.128
Albumin (g/dL)	3.07 ± 0.75	3.08 ± 0.79	3.06 ± 0.74	0.920
Hct (%)	32.36 ± 8.56	35.1 ± 10.63	31.70 ± 7.92	0.110
WBC (cells/mm^3^)	13,508.06 ± 7372.62	15,122.4 ± 7906.19	13,123.69 ± 7236.48	0.278
Neutrophil (%)	81.57 ± 12.55	76.70 ± 18.28	82.73 ± 10.57	0.053
Platelet (cells/mm^3^)	221,885.60 ± 138,754.90	231,950.00 ± 107,255.50	219,489.30 ± 145,700.3	0.720

Abbreviations: BUN, blood urea nitrogen; eGFR, estimated glomerular filtration rate; Hct, hematocrit; WBC, white blood cell.

**Table 3:** Echocardiography findings.

Parameter	Mean ± SD			
	Total (*n* = 104)	RV dysfunction (*n* = 20)	No RV dysfunction (*n* = 84)	*p* value
LV end diastolic dimension (mm)	39.95 ± 7.17	37.68 ± 6.07	40.49 ± 7.34	0.116
LV mass index (g/m^2^)	97.32 ± 29.12	88.97 ± 25.17	99.31 ± 29.77	0.155
LV ejection fraction (%)	61.38 ± 11.98	50.99 ± 14.30	63.73 ± 10.09	< 0.001
Left atrial volume index (mL/m^2^)	34.37 ± 12.76	37.74 ± 12.21	33.63 ± 12.83	0.218
Diastolic function, *n* (%)				0.002
Normal	3 (2.9)	0	3 (3.6)	
Abnormal relaxation	64 (61.5)	7 (35.0)	57 (67.9)	
Pseudonormalization	25 (24.0)	6 (30.0)	19 (22.6)	
Uncertain	12 (11.5)	7 (35.3)	5 (5.9)	
Right ventricular systolic function				
TAPSE (mm)	20.42 ± 3.09	13.97 ± 3.09	21.96 ± 3.44	< 0.001
S′ (cm/sec)	12.50 ± 3.66	7.34 ± 1.33	13.72 ± 2.88	< 0.001
RV FAC (%)	44.20 ± 10.66	28.47 ± 6.34	47.94 ± 7.622	< 0.001
TR V max (mmHg)	31.09 ± 11.31	32.72 ± 11.13	30.67 ± 11.45	0.595
RVSP (mmHg)	43.00 ± 12.01	44.99 ± 13.69	42.49 ± 11.67	0.543
Mean pulmonary arterial pressure by Abbas's formula (mmHg)	26.82 ± 1.23	34.74 ± 2.31	25.45.27 ± 6.61	0.006
Right atrial pressure (mmHg)	11.28 ± 2.53	11.07 ± 3.11	11.33 ± 2.39	0.688
IVC diameter (cm)	1.04 ± 0.54	1.37 ± 0.67	1.41 ± 0.50	0.754

*Note:* S′, tissue Doppler-derived tricuspid lateral annular systolic velocity.

Abbreviations: IVC, inferior vena cava; LV, left ventricular; RV FAC, right ventricular fractional area change; RVSP, right ventricular systolic pressure; TAPSE, tricuspid annular plane systolic excursion; TRV max, tricuspid regurgitant velocity max.

**Table 4:** Prognostic factors associated with 28-day in-hospital mortality.

Prognostics	Death (*n* = 19)	Alive (*n* = 85)	Crude hazard ratio	*p* value
DM, *n* (%)	7 (36.8)	48 (56.5)	0.50	0.142
CKD, *n* (%)	4 (21.1)	24 (28.2)	0.70	0.522
Cirrhosis, *n* (%)	0 (0.0)	9 (10.6)	NA	0.206
Septic shock, *n* (%)	10 (52.6)	22 (25.9)	2.60	0.038
Mechanical ventilation, *n* (%)	14 (73.7)	28 (32.9)	4.68	0.003
ARDS, *n* (%)	1 (5.3)	3 (3.53)	1.67	0.616
SOFA score, mean ± SD	6.26 ± 4.01	4.72 ± 2.57	1.20	0.021
APACHE II score, mean ± SD	17.58 ± 6.45	14.47 ± 5.18	1.09	0.027
Inotropic drugs used, *n* (%)	9 (47.4)	20 (23.5)	2.39	0.058
Acute kidney injury, *n* (%)	12 (63.2)	48 (56.5)	1.33	0.549
Bicarbonate (mmol/L), mean ± SD	20.53 ± 3.72	20.42 ± 5.36	1.00	0.923
pH, mean ± SD	7.37 ± 0.10	7.37 ± 0.12	1.10	0.961
Maximal blood lactate (mmol/L), mean ± SD	5.11 ± 4.91	3.91 ± 3.41	1.09	0.132
High sense troponin (ng/mL), mean ± SD	100.8 ± 117.18	305.35 ± 576.35	0.99	0.597
Left ventricular ejection fraction (%)	58.68 ± 14.12	61.95 ± 11.49	0.98	0.273
Right ventricular dysfunction, *n* (%)				< 0.001
No	8 (42.1)	76 (89.4)	1.00	
Yes	11 (57.9)	9 (10.6)	7.86	

*Note:* APACHE II score, Acute Physiology and Chronic Health Evaluation II.

Abbreviations: ARDS, acute respiratory distress syndrome; CKD, chronic kidney disease; DM, diabetes mellitus; SOFA score, Sequential Organ Failure Assessment score.

**Table 5:** Results of cox proportional hazard regression analysis.

Prognostics	Crude hazard ratio	*p* value	Adjusted hazard ratio	95% CI	*p* value
RV dysfunction					
No	1		1		
Yes	7.86	< 0.001	5.53	1.98–15.42	0.001
DM	0.50	0.142	0.86	0.32–2.28	0.757
Septic shock	2.60	0.038	5.34	0.61–4.10	0.149
Mechanical ventilation	4.68	0.003	2.18	0.68–6.99	0.189
APACHE II score ≥ 15	2.56	0.057	1.29	0.44–3.82	0.647
Maximal blood lactate	1.09	0.132	0.99	0.85–1.15	0.854
Inotropic drugs used	2.39	0.058	0.19	0.02–1.77	0.143
SOFA score	1.20	0.021	1.10	0.88–1.37	0.401

*Note:* APACHE II score, Acute Physiology and Chronic Health Evaluation II; SOFA score, Sequential Organ Failure Assessment score.

Abbreviations: DM, diabetes mellitus; RV dysfunction, right ventricular dysfunction.

**Table 6:** Secondary outcomes.

Secondary outcomes	RV dysfunction (*n* = 20)	No RV dysfunction (*n* = 84)	Mean difference	95% CI	*p* value
Blood lactate (mmol/L), mean ± SD	5.72 ± 4.95	3.74 ± 3.29	1.98	0.15–3.81	0.034
Length of ICU stay (days), mean ± SD	6.50 ± 2.86	2.84 ± 1.56	3.67	0.58–6.75	0.020
Duration of mechanical ventilation (days), mean ± SD	5.80 ± 6.07	2.78 ± 6.07	3.03	0.15–6.20	0.062

Abbreviations: ICU, intensive care unit; RV, right ventricular.

## Data Availability

The data that support the findings of this study are available from the corresponding author upon reasonable request.

## References

[1] Jawad I., Lukšić I., Rafnsson S. B. (2012). Assessing Available Information on the Burden of Sepsis: Global Estimates of Incidence, Prevalence and Mortality. *Journal of Global Health*.

[2] Rudd K. E., Johnson S. C., Agesa K. M. (2020). Global, Regional, and National Sepsis Incidence and Mortality, 1990–2017: Analysis for the Global Burden of Disease Study. *The Lancet*.

[3] Vincent J.-L., Marshall J. C., Ñamendys-Silva S. A. (2014). Assessment of the Worldwide Burden of Critical Illness: The Intensive Care Over Nations (ICON) Audit. *The Lancet Respiratory Medicine*.

[4] Beesley S. J., Weber G., Sarge T. (2018). Septic Cardiomyopathy. *Critical Care Medicine*.

[5] Winkelhorst J. C., Bootsma I. T., Koetsier P. M., de Lange F., Boerma E. C. (2020). Right Ventricular Function and Long-Term Outcome in Sepsis: A Retrospective Cohort Study. *Shock*.

[6] Vallabhajosyula S., Kumar M., Pandompatam G. (2017). Prognostic Impact of Isolated Right Ventricular Dysfunction in Sepsis and Septic Shock: An 8-Year Historical Cohort Study. *Annals of Intensive Care*.

[7] Padang R., Chandrashekar N., Indrabhinduwat M. (2020). Aetiology and Outcomes of Severe Right Ventricular Dysfunction. *European Heart Journal*.

[8] Vallabhajosyula S., Shankar A., Vojjini R. (2021). Impact of Right Ventricular Dysfunction on Short-Term and Long-Term Mortality in Sepsis: A Meta-Analysis of 1373 Patients. *Chest*.

[9] Singer M., Deutschman C. S., Seymour C. W. (2016). The Third International Consensus Definitions for Sepsis and Septic Shock (Sepsis-3). *JAMA*.

[10] Levey A. S., Stevens L. A., Schmid C. H. (2009). A New Equation to Estimate Glomerular Filtration Rate. *Annals of Internal Medicine*.

[11] Rudski L. G., Lai W. W., Afilalo J. (2010). Guidelines for the Echocardiographic Assessment of the Right Heart in Adults: A Report From the American Society of Echocardiography. *Journal of the American Society of Echocardiography*.

[12] Murphy E., Shelley B. (2019). Clinical Presentation and Management of Right Ventricular Dysfunction. *BJA Education*.

[13] Mekontso Dessap A., Boissier F., Charron C. (2016). Acute Cor Pulmonale During Protective Ventilation for Acute Respiratory Distress Syndrome: Prevalence, Predictors, and Clinical Impact. *Intensive Care Medicine*.

[14] Spapen H., Vincken W. (1992). Pulmonary Arterial Hypertension in Sepsis and the Adult Respiratory Distress Syndrome. *Acta Clinica Belgica*.

[15] Redl G., Germann P., Plattner H., Hammerle A. (1993). Right Ventricular Function in Early Septic Shock States. *Intensive Care Medicine*.

[16] Petit M., Jullien E., Vieillard-Baron A. (2021). Right Ventricular Function in Acute Respiratory Distress Syndrome: Impact on Outcome, Respiratory Strategy and Use of Veno-Venous Extracorporeal Membrane Oxygenation. *Frontiers in Physiology*.

[17] Ventetuolo C. E., Klinger J. R. (2014). Management of Acute Right Ventricular Failure in the Intensive Care Unit. *Annals of the American Thoracic Society*.

[18] Lakshmanadoss U., Levitan B. M., Hsi D. H. (2011). Right Ventricle Failure in Sepsis: A Case Report. *Cardiology Research*.

[19] Zochios V., Parhar K., Tunnicliffe W., Roscoe A., Gao F. (2017). The Right Ventricle in ARDS. *Chest*.

[20] Innocenti F., Palmieri V., Stefanone V. T. (2020). Epidemiology of Right Ventricular Systolic Dysfunction in Patients With Sepsis and Septic Shock in the Emergency Department. *Internal and Emergency Medicine*.

[21] Maeder M., Fehr T., Rickli H., Ammann P. (2006). Sepsis-Associated Myocardial Dysfunction: Diagnostic and Prognostic Impact of Cardiac Troponins and Natriuretic Peptides. *Chest*.

[22] Tongyoo S., Permpikul C., Lertsawangwong S. (2011). Right Ventricular Dysfunction in Septic Shock. *Journal of the Medical Association of Thailand*.

[23] Furian T., Aguiar C., Prado K. (2012). Ventricular Dysfunction and Dilation in Severe Sepsis and Septic Shock: Relation to Endothelial Function and Mortality. *Journal of Critical Care*.

[24] Bendary A., Said H., Elemary M., Mahrous M. (2022). Right Ventricular Function as a Predictor of Short-Term Mortality in Patients With Sepsis and Septic Shock: An Observational Study. *The Egyptian Heart Journal*.

[25] Lanspa M. J., Cirulis M. M., Wiley B. M. (2021). Right Ventricular Dysfunction in Early Sepsis and Septic Shock. *Chest*.

[26] Orde S. R., Pulido J. N., Masaki M. (2014). Outcome Prediction in Sepsis: Speckle Tracking Echocardiography Based Assessment of Myocardial Function. *Critical Care*.

[27] Liu Z., Meng Z., Li Y. (2019). Prognostic Accuracy of the Serum Lactate Level, The SOFA Score and the qSOFA Score for Mortality Among Adults With Sepsis. *Scandinavian Journal of Trauma, Resuscitation and Emergency Medicine*.

[28] Williams T. A., Ho K. M., Dobb G. J., Finn J. C., Knuiman M., Webb S. A. R. (2010). Effect of Length of Stay in Intensive Care Unit on Hospital and Long-Term Mortality of Critically Ill Adult Patients. *British Journal of Anaesthesia*.

[29] Mor-Avi V., Lang R. M., Badano L. P. (2011). Current and Evolving Echocardiographic Techniques for the Quantitative Evaluation of Cardiac Mechanics: ASE/EAE Consensus Statement on Methodology and Indications Endorsed by the Japanese Society of Echocardiography. *European Journal of Echocardiography*.

[30] Lang R. M., Badano L. P., Mor-Avi V. (2015). Recommendations for Cardiac Chamber Quantification by Echocardiography in Adults: An Update From the American Society of Echocardiography and the European Association of Cardiovascular Imaging. *European Heart Journal-Cardiovascular Imaging*.

[31] Konstam M. A., Kiernan M. S., Bernstein D. (2018). Evaluation and Management of Right-Sided Heart Failure: A Scientific Statement From the American Heart Association. *Circulation*.

